# Variation in Temperature Dependences across Europe Reveals the Climate Sensitivity of Soil Microbial Decomposers

**DOI:** 10.1128/aem.02090-22

**Published:** 2023-05-10

**Authors:** Carla Cruz-Paredes, Dániel Tájmel, Johannes Rousk

**Affiliations:** a Microbial Ecology, Department of Biology, Lund University, Lund, Sweden; Colorado School of Mines

**Keywords:** temperature relationships, climate change, microbial community composition, microbial growth, soil respiration, temperature sensitivity

## Abstract

Temperature is a major determinant of biological process rates, and microorganisms are key regulators of ecosystem carbon (C) dynamics. Temperature controls microbial rates of decomposition, and thus warming can stimulate C loss, creating positive feedback to climate change. If trait distributions that define temperature relationships of microbial communities can adapt to altered temperatures, they could modulate the strength of this feedback, but if this occurs remains unclear. In this study, we sampled soils from a latitudinal climate gradient across Europe. We established the temperature relationships of microbial growth and respiration rates and used these to investigate if and with what strength the community trait distributions for temperature were adapted to their local environment. Additionally, we sequenced bacterial and fungal amplicons to link the variance in community composition to changes in temperature traits. We found that microbial temperature trait distributions varied systematically with climate, suggesting that an increase in mean annual temperature (MAT) of 1°C will result in warm-shifted microbial temperature trait distributions equivalent to an increase in temperature minimum (*T*_min_) of 0.20°C for bacterial growth, 0.07°C for fungal growth, and 0.10°C for respiration. The temperature traits for bacterial growth were thus more responsive to warming than those for respiration and fungal growth. The microbial community composition also varied with temperature, enabling the interlinkage of taxonomic information with microbial temperature traits. Our work shows that the adaptation of microbial temperature trait distributions to a warming climate will affect the C-climate feedback, emphasizing the need to represent this to capture the microbial feedback to climate change.

**IMPORTANCE** One of the largest uncertainties of global warming is if the microbial decomposer feedback will strengthen or weaken soil C-climate feedback. Despite decades of research effort, the strength of this feedback to warming remains unknown. We here present evidence that microbial temperature relationships vary systematically with environmental temperatures along a climate gradient and use this information to forecast how microbial temperature traits will create feedback between the soil C cycle and climate warming. We show that the current use of a universal temperature sensitivity is insufficient to represent the microbial feedback to climate change and provide new estimates to replace this flawed assumption in Earth system models. We also demonstrate that temperature relationships for rates of microbial growth and respiration are differentially affected by warming, with stronger responses to warming for microbial growth (soil C formation) than for respiration (C loss from soil to atmosphere), which will affect the atmosphere-land C balance.

## INTRODUCTION

Temperature is a dominant controller of biological process rates at all levels of biological organization (1). Thus, changes in thermal regimes will influence how C is processed through all ecosystems. Microorganisms dominate the metabolic activity underpinning decomposition and therefore constitute a major determinant of C fluxes in the biosphere. To accurately predict how warming will alter C dynamics we need to understand how microorganisms depend on temperature. Warmer temperatures will accelerate microbial respiration within the studied environment’s temperature range (2, 3). Consequently, it is generally expected that warming will stimulate losses of C into the atmosphere (4, 5) representing positive feedback to climate warming, accelerating change. Despite considerable scientific attention in recent decades, there are multiple gaps in our understanding of microbial responses to warming. For instance, by simultaneously determining microbial temperature dependences of both primary production and decomposition in running water ecosystems, it was shown that warming induced a shift toward more heterotrophy (5, 6), while other studies found similar responses of primary production and decomposition, resulting in no net change in metabolic balance (7). In soils, long-term warming experiments have shown that the initial increase in soil respiration diminishes with time, gradually recovering to ambient values (8–10). It has been suggested that this can be due to substrate depletion (8, 11, 12), changes due to plant feedbacks (12), shifts in microbial community composition (13), or microbial thermal adaptation (14–18). The existence of microbial thermal adaptation is of critical importance since it can modulate the positive feedback between climate warming and ecosystem CO_2_ release. In addition, it is unknown if microbial communities will share a temperature dependence for respiration (immediate C loss from soil) and for synthesis of microbial biomass (potential microbial-C input to soil) (19–21) and how these will respond to warming. This balance has implications for the fate of C as stored microbially derived C or atmospheric CO_2_ release (22).

Although the thermal adaptation of microbial respiration and microbial community growth has been studied along climate gradients (16, 17, 23–27), in laboratory experiments (28), and in field warming experiments (18, 29, 30), how and to what extent it will affect predictions of the microbial feedback to climate warming remains uncertain on a global scale (31). It is anticipated that the environmental temperature will determine the temperature sensitivity of the microbial community by selecting for a community with trait distributions adapted to facilitate rates of growth and respiration at the prevailing temperature regime (32, 33). Yet the strength by which trait distributions are shaped by the thermal regime, i.e., how much microbial temperature relationships shift per degree Celsius warmer temperature, has yet to be generalized.

At low temperatures, growth and activity rates are low, starting from zero at the apparent temperature minimum (*T*_min_). When temperatures increase above *T*_min_, rates also increase until a maximal rate at the optimum temperature (*T*_opt_). At temperatures exceeding *T*_opt_, rates will again decrease until zero is reached at an apparent maximum temperature (*T*_max_), altogether making up the temperature relationship for a community. The square root (or “Ratkowsky”) model is a simple model that can represent these microbial temperature relationships (23, 33–35). This model enables the estimation of useful indices that can be used to compare microbial community temperature trait distributions, including the lower temperature limit for activity (minimum temperature [*T*_min_]), the temperature for optimal rates (the optimum temperature [*T*_opt_]), and the upper temperature limit for activity (the maximum temperature [*T*_max_]). Any of these can be used to screen for changes or differences in temperature relationships, where higher values denote communities with warm-adapted traits, and lower values denote cold-adapted traits. The *T*_min_ can be determined with high accuracy, which is why it has been used as an effective index for temperature relationships in comparative assessments in very different ecosystems (26, 27, 30), making it possible to compare the level of the temperature adaptation for trait distributions for microbial growth and respiration (23, 33).

**FIG 1:**
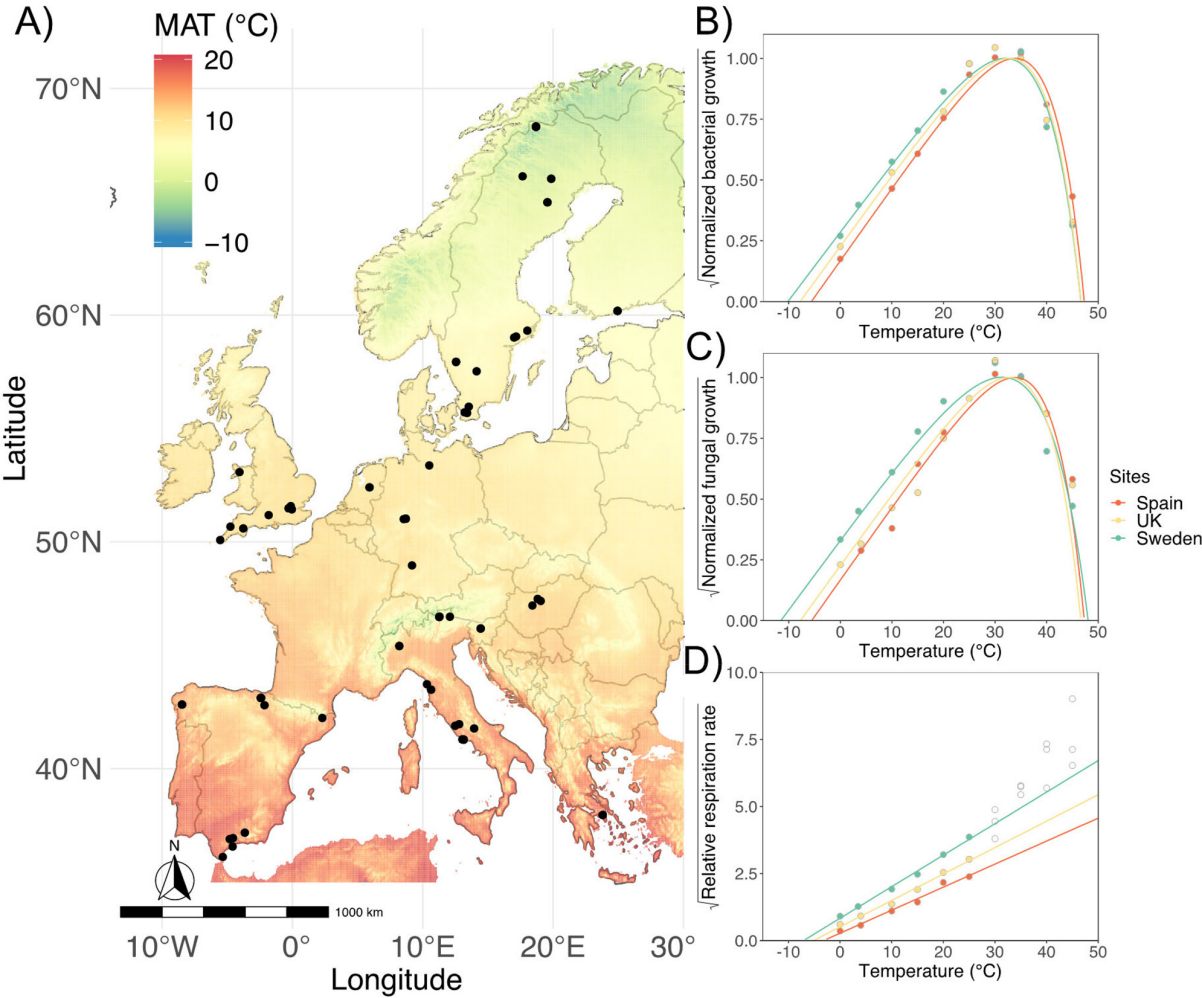
(A) Soil sampling points across a European gradient. Colors represent the historical mean annual temperatures (MAT) between 1970 and 2000. More information on the sampling sites is presented in Table S1. (B to D) Temperature relationships for square root transformed rates of (B) bacterial growth, (C) fungal growth, and (D) respiration rate in a comparison of three selected contrasting sites from the 72 collected across Europe (see Fig. 2). The orange site is in Spain with a MAT of 17.4°C, the yellow site is in the United Kingdom (UK) with a MAT of 10.3°C, and the green site is in Sweden with a MAT of 6.5°C. Bacterial and fungal growth rates are normalized to the optimum temperature (*T*_opt_) for growth. Due to normalization, the microbial growth is unit-less. Fitted curves are based on the Ratkowsky model. Below the *T*_opt_, square root transformed rates showed a linear response to the screening temperature. Unlike bacterial and fungal growth, the respiration rate did not reach a temperature optimum or maximum in the studied incubation temperature interval. Therefore, the simplified square root relationship was used (equation 2; see Materials and Methods). The open circles in panel D indicate the excluded data points for the linear fitting.

In this study, we set out to define the variance of temperature relationships of both microbial growth and soil respiration at a continental scale to determine if and how microbial communities’ distributions of temperature traits are adapted to the environmental temperature regime. Since latitudinal gradients have stable and large temperature differences, the assembly of microbial communities should have had sufficient time for ecological and evolutionary processes to act, allowing us to distinguish if soil microbial communities’ temperature relationships are matched to the local climate. To this end, we investigated a latitudinal gradient across Europe with 72 sites that spanned through a comprehensive gradient of mean annual temperatures (MAT) from −3.1 to 18.3°C (Fig. 1A). The surveyed soils were intentionally selected to include wide ranges of soil organic matter (SOM), pH, and land uses in all climates. We resolved if thermal trait distributions that define the temperature relationships for bacterial growth, fungal growth, and respiration were adapted to the climate of the site. We did this by determining the temperature relationships for growth rates of bacteria and fungi, along with soil respiration rate in different sites. We estimated the indices for the temperature relationships for microbial growth and respiration, including *T*_min_, which were used for a comparative assessment of the variation of microbial temperature relationships across Europe, where higher values denoted communities with warm-adapted trait distributions (19, 30). Additionally, we determined the consequences of the temperature sensitivity (*Q*_10_) for microbial growth and respiration resulting from these differences. The indices for the temperature relationships for each process were then related to environmental temperatures (MAT), allowing us to quantify the strength of the dependence of microbial temperature relationships on the environmental temperature regime. To interlink the microbial community composition to the differences in microbial temperature traits, we sequenced bacterial (16S) and fungal (internal transcribed spacer [ITS]) amplicons from the different sites to assess the α- and β-diversity variance across the latitudinal gradient.

## RESULTS

**FIG 2:**
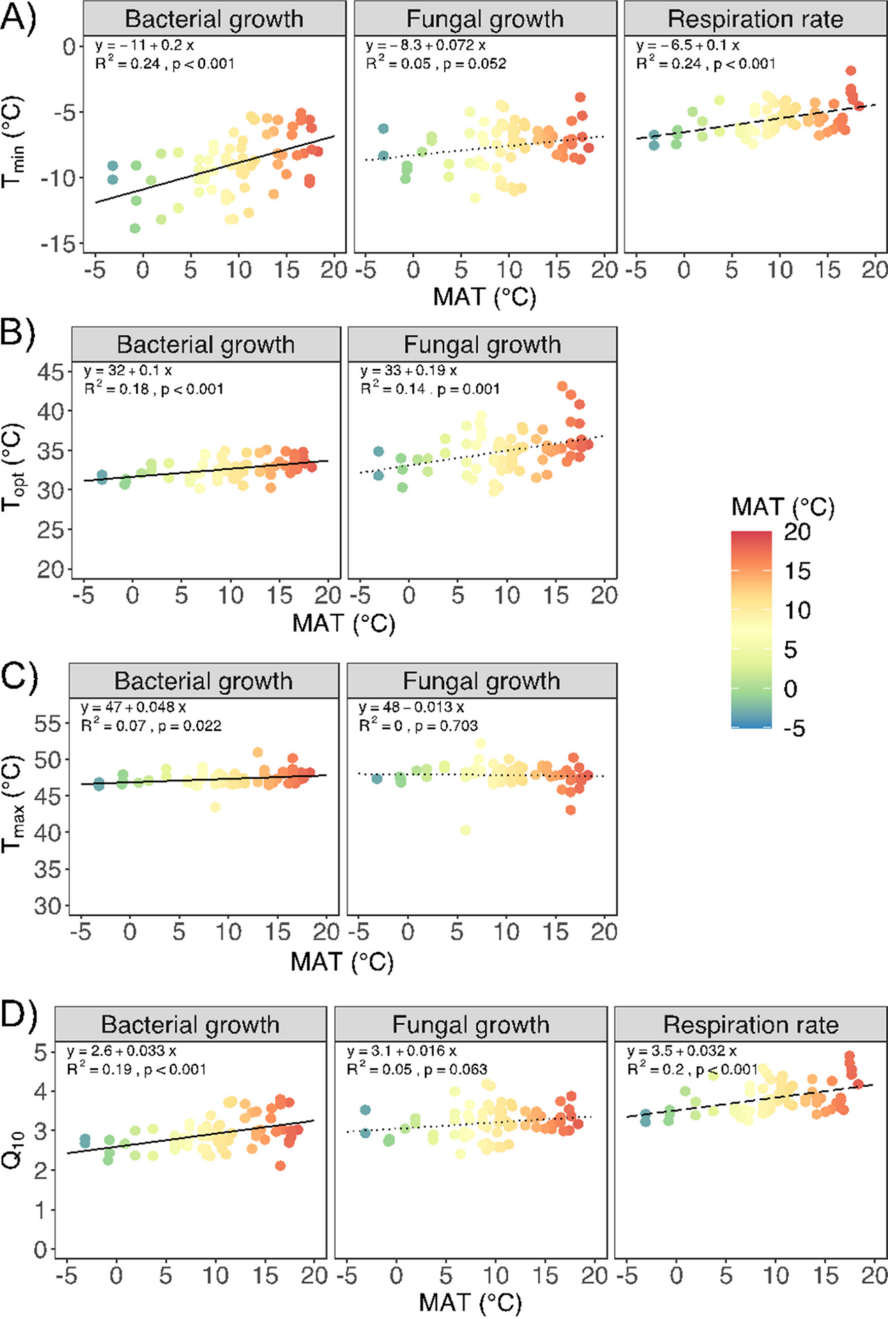
(A) Minimum temperature (*T*_min_) for bacterial growth, fungal growth, and respiration rate; (B) optimum temperature (*T*_opt_) for bacterial and fungal growth; (C) maximum temperature (*T*_max_) for bacterial and fungal growth; (D) *Q*_10(5-15°C)_ for bacterial growth, fungal growth, and respiration rate regressed against mean annual temperature (MAT). Colors represent the MAT across the European gradient. Lines represent linear regression fits; equations, *R*^2^ values, and *P* values for each linear regression are reported in each graph.

We characterized the temperature relationships for bacterial growth, fungal growth, and respiration for all sites (see Table S1 in the supplemental material). Temperature relationships varied systematically with MAT, where higher MAT resulted in microbial communities with warm-adapted trait distributions for growth and respiration (Fig. 1 and 2). The modeled temperature relationships for microbial growth rate, started at a minimum temperature for growth (*T*_min_) (Fig. 1B and C) between −14 and −5°C for bacteria and from −11 to −4°C for fungi (Fig. 2A). From *T*_min_, microbial growth continually increased with higher temperature until a maximal value at the optimum temperature (*T*_opt_) (Fig. 1B and C) of 30 to 35°C for bacterial growth and of 30 to 43°C for fungal growth (Fig. 2B). At temperatures above *T*_opt_, microbial growth rates decreased until they reached zero, thus defining the maximal temperature (*T*_max_) of the modeled temperature relationships (Fig. 1B and C) for growth, which varied between 43 and 51°C for bacteria and 40 to 52°C for fungi (Fig. 2C). Bacteria and fungi showed differences in *T*_opt_ and *T*_max,_ with fungi having wider ranges (Fig. 2B and C). The temperature relationships for respiration started at *T_min_* values between −8 and −2°C (Fig. 2A); rates increased with higher temperatures throughout the studied temperature interval without dropping during the brief assays (Fig. 1D). In general, sites with higher historical MAT had warm-adapted temperature trait distributions for bacterial growth and respiration (bacterial *F*_1,69_ = 22.1, *P* < 0.001; respiration *F*_1,70_ = 22.3, *P* < 0.001, where *F*-test statistic and the numbers are degrees of freedom), while for fungal growth there were only tendencies (*F*_1,70_ = 3.9, *P* = 0.052) (Fig. 2; Table S3). We observed a similar pattern for the *Q*_10(5-15°C)_ values (bacterial *F*_1,69_ = 16.9, *P* < 0.001; fungal *F*_1,70_ = 4.2, *P* = 0.04; respiration *F*_1,70_ = 24.4, *P* < 0.001), which increased in sites with higher MAT (Fig. 2D). Our results suggest that an increase in MAT of 1°C will result in warm-shifted microbial temperature relationships equivalent to an increase of *T*_min_ of 0.20°C for bacteria, 0.07°C for fungi, and 0.10°C for respiration. For the temperature sensitivity (*Q*_10_), an increase in MAT of 1°C will result in an increase of *Q*_10_ of 0.03 units for bacteria, 0.02 units for fungi, and 0.03 units for respiration.

Our survey was conducted during the summer and winter of 2019 and 2020. To control for the explanation that the determined microbial temperature relationships reflected the seasonal temperature at the time of sampling, we evaluated the effect of season and MAT through an analysis of covariance (ANCOVA). We found that only fungal *T*_min_ had a significant effect of season (*F*_1,68_ = 5.3, *P* = 0.03), due to lower values in the summer sampling compared to the winter (Fig. S1B). However, we found no significant effect of the interactions between season and MAT in any of the temperature relationship indices. In addition, we selected a subset of 10 soils from nearby sites that were sampled both in winter and summer (Table S2). A paired *t* test revealed that the soils sampled in both summer and winter have indistinguishable temperature relationships for bacteria (*T*_min_ *t*_3_ = −1.2, *P* = 0.33; *T*_opt_ *t*_3_ = −1.5, *P* = 0.23; *T*_max_ *t*_3_ = −1.1, *P* = 0.36, where *t* is the *t*-test statistic and the number is the degrees of freedom), fungi (*T*_min_ *t*_4_ = −1.6; *P* = 0.18; *T*_opt_ *t*_4_ = −1.1; *P* = 0.34; *T*_max_ *t*_4_ = 1.4; *P* = 0.25), and respiration (*T*_min_ *t*_4_ = −1.0, *P* = 0.39), respectively. Finally, we found no indication that the annual amplitude in environmental temperature variation could explain microbial adaptation to a stronger variation, since there was no significant relationship of the difference between summer and winter temperatures and the width of the temperature relationships (*T*_max_ − *T*_min_) for bacteria or fungi (Fig. S2).

To determine what environmental conditions had given rise to the variation in microbial temperature relationships, the indices for temperature relationships (*T*_min_, *T*_opt_, *T*_max_) were regressed against both mean annual summer and mean annual winter temperatures (Table S3). These comparisons indicated steeper responses to summer temperatures than to winter temperatures (Table S3). Other environmental variables were also regressed against the indices for temperature relationships (Table S4). We found that pH and SOM were positively related with some of the indices for microbial growth and respiration. Fungal *T*_min_ was more strongly linked to soil pH (*R*^2^ = 0.09) than to MAT, while fungal *T*_opt_ was better explained by pH (*R*^2^ = 0.26) and SOM (*R*^2^ = 0.25) (Table S4). The multiple regression analysis (Table S5) showed that MAT was the only significant explanatory variable for the *T*_min_s of bacterial growth (*p*_MAT_ (*p*-value for MAT variable) < 0.001; model *F*_4,66_ = 5.5, *R*^2^= 0.25, *P* = 0.001) and respiration (*p*_MAT_ = 0.001; model *F*_4,67_ = 6.2, *R*^2^= 0.27, *P* < 0.001), while there was no significant predictor for fungal growth *T*_min_. For bacterial *T*_opt_, both SOM and MAT were significant predictors (*p*_SOM_ (*p*-value for SOM variable) = 0.02; *p*_MAT_ = 0.02; model *F*_4,66_ = 5.3, *R*^2^ = 0.24, *P* = 0.001), while we did not find any significant predictors for fungal growth *T*_opt_ or bacterial and fungal growth *T*_max_s (Table S5). Finally, we found no correlation between C availability estimated as the rate of microbial C use per SOM and MAT (Pearson’s correlation [corr] = 0.18, *P *= 0.16; Fig. S3).

**FIG 3:**
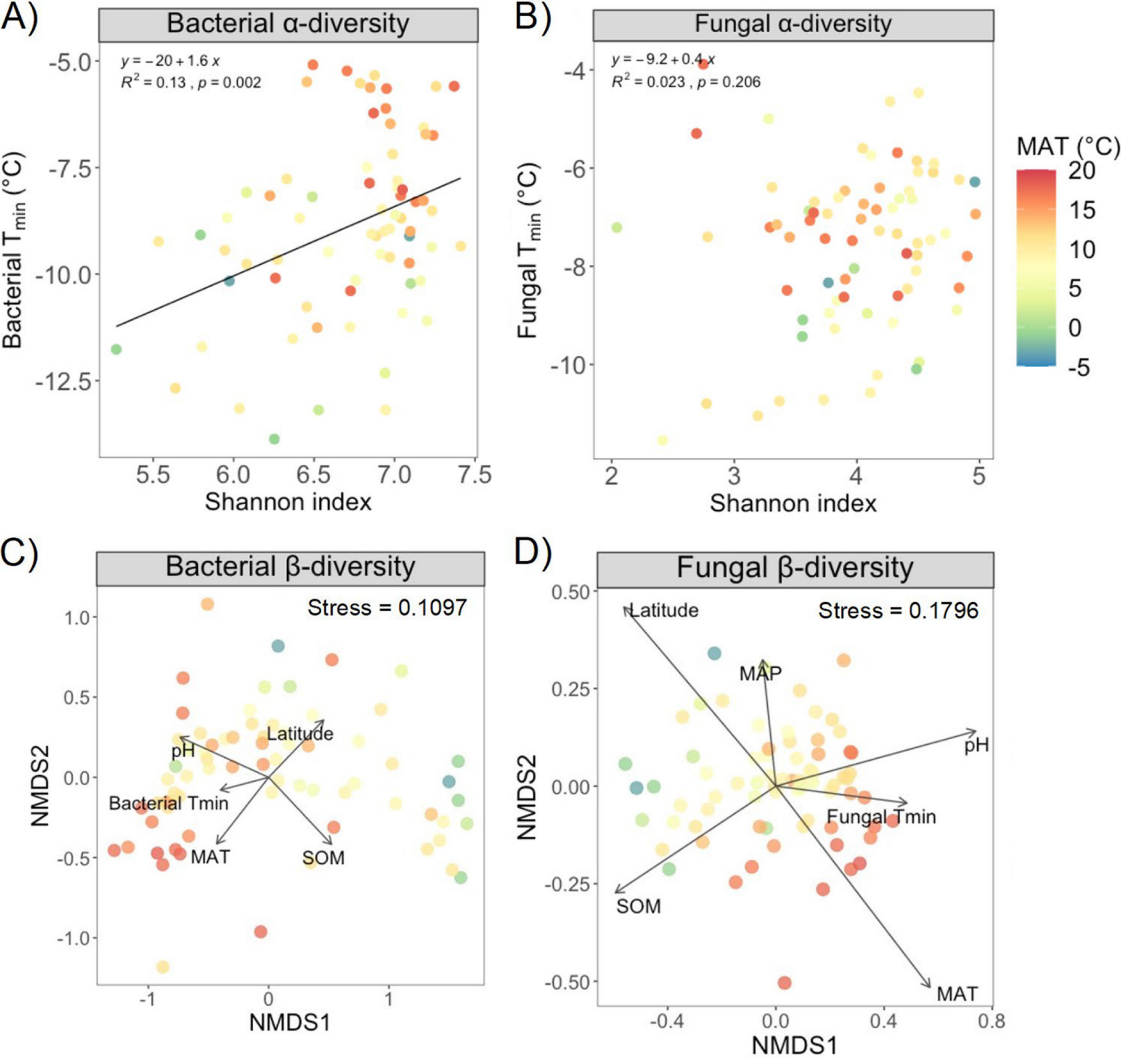
(A) Linear regression between the bacterial alpha diversity evaluated as the Shannon index and the bacterial minimum temperature (*T*_min_). (B) Linear regression between the fungal alpha diversity evaluated as the Shannon index and the fungal minimum temperature (*T*_min_). Lines represent linear regression fits; equations, *R^2^* values, and *P* values for each linear regression are reported in each graph. (C and D) NMDS plots based on Bray-Curtis dissimilarity measures showing (C) the bacterial community composition and (D) the fungal community composition in the different sites across the temperature gradient. Arrows represent environmental variables that correlate significantly (*P* < 0.05) with the variance in the communities’ composition calculated with the function envfit from the vegan package; the length of the arrow represents the *R*^2^ value (see Table S8). Colors represent MATs across the European gradient.

To investigate if the microbial community’s taxonomic composition was linked to differences in thermal trait distributions of the microbial community, we regressed the bacterial and fungal growth *T*_min_s against bacterial and fungal α-diversity (Table S6). The bacterial growth *T*_min_ was positively related to the bacterial α-diversity (*F*_1,69_ = 10.6, *R*^2^ = 0.13, *P* = 0.002; Fig. 3A), while no patterns could be discerned for fungal growth *T*_min_ (*F*_1,70_ = 1.6, *R*^2^ = 0.02, *P*= 0.21; Fig. 3B). MAT and soil pH were also positively related to bacterial α-diversity, while SOM was negatively related (Table S6). Moreover, soil pH was also positively related to fungal α-diversity (Table S6). Furthermore, the envfit function showed that the bacterial community composition was significantly correlated with the bacterial growth *T*_min_ (*R*^2^ = 0.17, *P* = 0.002; vector in Fig. 3C). Similarly, the fungal community composition was also significantly correlated with the variation in fungal growth *T*_min_ (*R*^2^ = 0.24, *P* = 0.001; vector in Fig. 3D). We identified 1,501 bacterial amplicon sequence variants (ASVs) that correlated significantly with high *T*_min_ values and 2,171 ASVs that correlated with low *T*_min_ values (Supplemental File 1; Table S7). For fungi, we found 270 ASVs that correlated significantly with high *T*_min_ values and 425 ASVs that correlated with low *T*_min_ values (Appendix 1; Table S7).

Several additional environmental factors were also significantly correlated with the microbial community composition (Table S8). The results indicated that pH, SOM, MAT, and latitude were highly correlated with both the bacterial and the fungal community composition (vectors in Fig. 3C, D).

## DISCUSSION

Previous large-scale surveys attempting to link microbial thermal adaptation to climatic differences have found differences between sites, but most of them have been unable to demonstrate a systematic link between the temperature relationships of microbial communities with differences in environmental temperatures (16, 17, 24). For instance, temperature sensitivities of acidic boreal forest floors have been found to diverge from both arctic (cooler) and tropical (warmer) environments (24), and while biomass-specific respiration rates with excess substrate have been found to decrease in warmer environments, temperature sensitivities (*Q*_10_s) were independent of environmental MAT in a global dryland survey (16) and in a geographic survey across the United States (17). Only a recent large-scale study in China was able to demonstrate the link between temperature relationships for respiration and MAT (36). In contrast, in our study, we demonstrated that soil microbial temperature relationships for both growth and respiration varied systematically with environmental temperatures at a continental scale, where higher MATs were associated with microbial communities with warm-adapted trait distributions for both growth and respiration. This variance in temperature relationships covering a wide MAT range from −3.1 to 18.3°C across Europe also quantified the strength with which the historical temperature regime had formed the microbial trait distributions for temperature. Using the obtained relationships in a space-for-time substitution, our results suggest that an increase in MAT of 1°C will result in warm-shifted microbial temperature trait distributions equivalent to an increase in *T*_min_ of 0.20°C (and *Q*_10(5-15°C)_ of 0.03 units) for bacterial growth, 0.07°C (and *Q*_10(5-15°C)_ increase of 0.02 units) for fungal growth, and 0.10°C (and *Q*_10(5-15°C)_ increase of.03 units) for respiration. This provides evidence for microbial communities with temperature traits adapted to the local environment for microbial growth and respiration and is the first assessment over a continental scale. Previous studies of altitudinal and regional climatic gradients and field warming experiments have estimated that *T*_min_ for bacterial growth and respiration increases by 0.2 to 0.8°C per 1°C increase in MAT (23, 25, 26, 30, 36, 37). This closely corresponds with the estimates in this study. Similarly, previous assessments of temperature gradients in Antarctic soils (26) and altitudinal gradients in tropical soils (25) found that *Q*_10_ increased by 0.03 and 0.05 units per 1°C temperature increase, respectively. Therefore, we can extend and refine the previous observations made across temperature gradients at smaller spatial scales to a continental-scale temperature gradient. The temperature relationships for bacterial growth had a stronger response to MAT than those for respiration or fungal growth. Consequently, our results suggest that the adaptation of bacterial temperature trait distributions will be more responsive to warming than those for respiration or fungal growth. It has been suggested that fungi are generally more resistant than bacteria to environmental changes such as changes in pH (38), and moisture (39), presumably due to their physiological plasticity. We also note that the method used to estimate fungal growth rates in intact soil samples *ex situ* included free-living fungi and excluded any contribution by symbiotic fungi that relied on, e.g., an intact plant host C supply (40). While the few attempts to estimate temperature relationships for mycorrhizal fungi suggest that they match those of saprotrophic fungi from the same environment (41), it is possible that the close physiological dependence that mycorrhizal fungi have on plants would differentiate them (42). In the case of respiration, it is a broad microbial function contributed to by both bacterial and fungal communities, which can increase as a response to both physiological stress and enhanced conditions for microbial growth (43–45), ambiguities that may weaken the link to climate temperatures compared to those for microbial growth.

Soil sampling was conducted during summer and winter; however, the determined microbial temperature relationships did not reflect the seasonal temperature at the time of sampling, since sites sampled in both winter and summer had indistinguishable temperature relationships (as revealed by the paired *t* test and Fig. S1). Our results therefore suggest that the continental patterns for microbial temperature trait adaptation were stable across seasonal changes in temperature. Additionally, the indices for microbial temperature trait distributions determined had steeper dependences on summer temperatures than winter temperatures, consistent with previous suggestions that warm periods dominate the environmental control of microbial temperature relationships. That is, periods during which temperatures reach values close to or exceeding the microbial *T*_opt_ are more important for determining the soil microbial temperature traits (27, 29, 37). Further, some sites had larger seasonal variation in temperature. This could have selected for wider temperature relationships in seasonally variable sites. To test for such a link, we calculated the difference between summer and winter temperatures and the width of the temperature relationships (*T*_max_ − *T*_min_) for bacteria and fungi. However, the width of temperature relationships was not systematically affected by the seasonal variation in temperature in the studied survey (Fig. S2).

Other environmental variables such as pH and SOM were also correlated with the temperature relationships. Previous studies have shown that temperature and precipitation are important factors that control soil pH (46, 47). Temperature mainly affects the rock weathering rate, while precipitation affects material flow. Additionally, along with climate, vegetation and its productivity will also play an important role in the regulation of soil pH (48). In the surveyed soils, intentionally selected to include wide ranges of SOM, pH, and land uses in all climates, environmental temperatures explained a dominant proportion of the microbial temperature trait variation, having stronger relationships with microbial temperature trait indices than other variables. This was also confirmed by a multiple regression analysis, which showed that climate temperature was the strongest factor shaping the variation in microbial temperature trait distributions for bacterial growth and respiration. However, this was not true for the temperature traits of fungal growth, since the measured variables did not significantly explain the variation in the fungal temperature relationship indices. This suggested that the variation in fungal temperature relationships was more strongly shaped by environmental variables not measured in this study, such as plant productivity, changes in plant community composition, or plant diversity (49–51). Moreover, the influence of substrate availability on temperature relationships is a topic that has received a lot of attention (52). It has widely been assumed that substrate independence is needed to isolate the temperature relationship from the influence of variable substrate availability (17, 18, 53). However, a recent study found that the temperature sensitivity was independent of the difference in chemical recalcitrance or C quality (54). In our study, using the rate of microbial C use per SOM as a proxy for C quality, we found no correlation between MAT and C availability in the different sites (Fig. S3). This showed that the observed differences in microbial thermal trait distributions and temperature sensitivities were not explained by differences in C quality along the gradient.

Variance in the microbial community composition was linked to differences in microbial temperature trait distributions. These links suggest that the long-term adaptation of microbial temperature traits is likely to have arisen from differences in microbial community composition (13, 32). It has been argued that in response to changes in temperature in the short-term, microbes can respond with physiological adjustment and acclimate to new temperatures (18, 55). However, in the long-term, species that thrive at the new temperature will outcompete others, more likely resulting in species turnover to yield higher relative abundances and activities of warm-adapted species, translating to differences in the microbial community composition (13, 31, 32).

Several additional environmental factors were also correlated with the microbial β-diversity. The envfit test indicated that pH, SOM, and MAT were highly correlated with the bacterial and fungal community composition. Since wide ranges of soil properties and land uses were included in all climates, it was expected that taxonomic breadth would be extensive in each climate. This is consistent with earlier findings revealing that pH is a major driver for differences in bacterial communities (56–58), while the structure of fungal communities also correlated with other drivers, possibly via plant community differences (58, 59). It has also been shown that MAT can be a significant predictor of the β-diversity of bacterial and fungal communities (13, 60–63), which indicates that MAT can have an important role in structuring the microbial community composition. However, the sensitivity to detect MAT-associated differences in taxonomic composition is likely to be higher when ranges of other environmental factors powerfully regulating taxonomic composition are smaller, yielding a higher signal-to-noise ratio. Given the high level of microbial β-diversity in soil samples, with taxonomic overlap often being <1% in pairwise comparisons of samples along steep environmental gradients (56, 58, 64), it is likely that the list of taxa with warm-adapted temperature traits would be nonoverlapping at pH 4 and 8. Such patterns would compromise the ability of ordination techniques to capture the link between temperature traits and microbial taxa (65). This could be addressed by using a stratified sampling where study sites across a wide climate gradient are selected to have smaller ranges of pH and SOM.

We also tried to identify warm- and cold-adapted taxa by investigating which ASVs’ relative abundance was correlated with high *T*_min_ values for warm-adapted taxa and with low *T*_min_ values for cold-adapted taxa. We found several bacterial ASVs that correlated significantly with high *T*_min_ values and low *T*_min_ values. Using the database Microbe Atlas (66), we found some indications that cold-adapted ASVs have been found mainly in samples classified as forest and tundra soils, while warm-adapted ASVs have been mainly found in samples classified as farm, field, agricultural, and desert soils (Table S7). For fungi, we used the database GlobalFungi (67) and found some indications that warm-adapted ASVs were more frequently sampled in sites with a MAT higher than 4°C, while cold-adapted ASVs were more frequently found in sites with a MAT lower than 8°C (Table S7). However, these findings only reveal an initial qualitative exploration. To be able to identify warm- and cold-adapted ASVs, observations from different manipulative experiments and natural temperature gradients should be combined to identify “common denominators” or “bioindicator taxa” that respond similarly to changes in temperature (68).

Understanding how ecosystem C balances are related to environmental temperatures and to warming is crucial for generating robust predictions from coupled climate-C models. Currently, soil C models that form the basis of Earth system models (ESMs) (e.g., Roth C, Century, Daycent, Candy) used to advise the Intergovernmental Panel on Climate Change (IPCC), assume a single, global temperature dependence for all microbial processes that is universal for all climates (69–72). Here, we show that the temperature trait distributions for microbial growth and respiration are adapted to their environment and are thus climate specific. We also quantified the strength of this dependence. These differences in temperature trait distributions resulted in associated differences in temperature sensitivity (*Q*_10_) for microbial growth and respiration, where communities with warm-shifted temperature trait distributions in warmer environments had higher temperature sensitivities. This validated earlier reports from separate ecosystems (26, 30), refined the estimate of the strength of the dependence, and extended them to a continental scale. Therefore, the use of a static temperature rate modifier currently used in ESMs (69–72) will not correctly represent the variation in microbial temperature relationships across the globe or in response to climate change. Thus, soil C models need to incorporate the climate adaptation of microbial temperature traits into soil C climate feedback predictions. Moreover, we show that the temperature trait distributions for bacterial growth, fungal growth, and respiration rate are differentially adapted to temperature, requiring further revision of current soil C models, by delineating these different microbial processes. The partitioning of microbial-used C into growth, which potentially can be stored, and respiration, which is immediately lost to the atmosphere, has been shown to be a fundamentally decisive parameter that will define the long-term balance of C dynamics (20–22, 73). We showed that temperature relationships for microbial growth will be more responsive to changes in temperature than respiration and that bacterial decomposers will respond more strongly than fungi will, which may affect the persistence of microbial necromass in soil due to their contrasting life history strategies (22, 74). Several studies have found that fungi can accumulate more necromass in both agricultural and forest soils (75–78) and that bacterial residues have a faster turnover rate than fungal residues (79, 80), suggesting that fungi contribute more to soil C stabilization. Asymmetrical responses of the temperature relationships for microbial growth and respiration to warmer temperatures have also been observed in aquatic ecosystems, where respiration was found to be more responsive (5, 6). The outcome of the asymmetrical sensitivities to warmer temperatures of microbial temperature traits for growth and respiration, and between the growth of different microbial groups, will affect the atmosphere-land C balance and thus the land-ecosystem feedback to climate warming.

## MATERIALS AND METHODS

### Soil sampling and characterization.

Soils were sampled across a European gradient in 72 sites that differed in their climate and soil characteristics and land use. The surveyed soils were intentionally selected to include wide ranges of SOM, pH, and land uses in all climates. Coordinates of the different sites were used to obtain mean annual temperature (MAT) and mean annual precipitation (MAP) from the Lund University Data Guru (https://dataguru.lu.se/). For MAT, monthly mean historical temperatures of 10 years (2009 to 2018) were obtained from the WFDEI data set (81). For MAP, monthly mean historical precipitation levels of 10 years (2002 to 2011) were obtained from the CRUTS version 3.20 data set (82). A map to illustrate the temperature range and the sampling site locations was created in R version 4.0.3 (83) with the raster package (84) using historical climatic data (85).

Composite soil samples from each site were taken by sampling several soil pits with a spade from the upper 5 cm until reaching ca. 300 g of soil. Soils were collected between June 2019 and June 2020 in different seasons (Table S1). All samples were stored at 4°C. Soils were sieved (<4 mm), moisture adjusted to 50% water holding capacity (WHC), and subsequently left in the dark at room temperature (20°C) for 1 week before being processed. Soil pH and electrical conductivity (EC) were measured in a 1:5 (wt/vol) soil/water extraction (5 g soil plus 25 mL H_2_O) using electrodes (combined pH electrodes, Radiometer Analytical, France and 4520 conductivity, Jenway, England, respectively). Soil organic matter (SOM) was measured using a loss-on-ignition procedure (86). The maximum amount of water that soils could hold after gravity loss was measured to assess the WHC of the soils (87). Information about sampling location, collection date, environmental data, and soil characteristics can be found in Table S1.

### Microbial temperature dependences.

Subsamples of the soils were transferred to different vials. Each vial was exposed to 1 of 10 different temperatures from 0°C to 45°C in 5°C intervals in water baths. Respiration, bacterial growth, and fungal growth were measured simultaneously for the different soils and temperatures in independent vials (*n *= 10 for each soil and each process; see below and File S2). The exposure to different temperatures during the incubation step was always kept to a duration corresponding to approximately 2 h at 20 to 25°C for bacterial growth, adapting the time of the incubation period for the other temperatures (i.e., 1 h at 30°C, 4 h at 15°C, etc., to ensure a similar level of C use in all treatments; see File S2). Similarly, incubations for fungal growth and respiration corresponded to ca. 4 h and 18 h, respectively, at 20 to 25°C and adapted for other temperatures (i.e., 2 h and 6 h at 30°C, 8 h and 30 h at 15°C, etc.; see File S2). Within these time periods, no change in growth rates or respiration due to altered conditions occurred (88), with the exception of the direct temperature effect on rates.

### Microbial growth and respiration.

Bacterial growth was measured with ^3^H-leucine incorporation (89; see File S2). The amount of leucine incorporated into extracted bacteria (pmol leucine incorporated g^−1^ soil h^−1^) was used as a proxy for bacterial growth. Fungal growth was determined using ^14^C-acetate in the fungus-specific lipid ergosterol (38, 90; see File S2). The amount of acetate incorporated into extracted ergosterol (pmol acetate incorporated g^−1^ soil h^−1^) was used as a proxy for fungal growth. Soil respiration was measured as CO_2_ production using a gas chromatograph equipped with a methanizer and a flame ionization detector (YL6500 GC, YL Instruments, South Korea).

### Microbial community.

DNA was extracted from 250 mg of freeze-dried soil using Power Soil Pro kits (MoBio, Carlsbad, USA) following the manufacturer’s instructions. DNA concentration was determined using a NanoDrop spectrophotometer system (Thermo Scientific, Wilmington, NC, USA), and DNA extracts were sent to BGI Tech Solutions (Hong Kong, China) for amplicon sequencing according to their standard protocol (see File S2). For bacterial communities, the V3 to V4 region of the 16S gene was amplified using the primers 341F (5′-CCTAYGGGRBGCASCAG-3′) and 806R (5′-GGACTACNNGGGTATCTAAT-3′) (91). For fungal communities the ITS1 to ITS2 region was amplified using the primers ITS1 (5′-CTTGGTCATTTAGAGGAAGTAA-3′) (92) and ITS2 (5′-GCTGCGTTCTTCATCGATGC-3′) (93). All sequence data were processed using DADA2 and the DADA2 ITS Pipeline Workflow version 1.8 (94) to determine the amplicon sequence variants (ASVs).

### Data analyses.

Temperature dependences of microbial growth and respiration were modeled using the Ratkowsky model (34) according to equation 1.

(1) $$R^{\text{1/2}}=a\left(T\;-\;T_{\text{min}}\right)\times \left(1\;-\;e^{b\left(T-T_{\text{max}}\right)}\right)$$

where *R* is the rate of leucine incorporation, acetate incorporation, or respiration, *a* and *b* are slope parameters, *T* is the screening temperature (°C), *T*_min_ is the minimum temperature (the first *x* axis intercept), and *T*_max_ is the maximum temperature (the second *x* axis intercept). At temperatures below optimum (0 to 25°C) and for respiration rate, the simplified equation 2 was used.

(2) $$R^{\text{1/2}}=a\left(T\;-\;T_{\text{min}}\right)$$

where parameters are the same as in equation 1. Equation 2 was first used to estimate *T*_min_ and the slope *a*, which could be used as constants in equation 1 for the entire temperature interval. *T*_max_ was estimated with equation 1. With the derivative of equation 1, the optimum temperature for growth (*T*_opt_) was estimated. Values were then normalized to the maximum rate (rate at *T*_opt_).

The temperature coefficient (*Q*_10_) was estimated as an index for the temperature sensitivity, according to equation 3.

(3) $$Q_{\text{10}}={\left[a\left(\left(T\;+\;10\right)\;-\;T_{\text{min}}\right)\right]}^{2}∕{\left[a\left(T\;-\;T_{\text{min}}\right)\right]}^{2}$$

where *Q*_10_ indicates how the rate (*R*) changes with a difference of 10°C. Parameters are as defined in equations 1 and 2, and the temperature interval 5 to 15°C was used.

To understand the relationship between the temperature regime and the temperature relationships and temperature sensitivity calculated, we regressed the indices for temperature relationships (*T*_min_, *T*_opt_, *T*_max_) and the temperature sensitivity (*Q*_10_) against soil MAT. Additionally, the temperature relationship indices were regressed with the mean temperature of the warmest month (summer temperature) and the mean temperature of the coldest month (winter temperature). The difference between summer and winter temperatures was also regressed against the width of the temperature relationships (*T*_max_ − *T*_min_) (see File S2 for details). To control for the seasonal temperature at the time of sampling, we studied the effect of the season on the regression between *T*_min_, *T*_opt_, *T*_max_, and MAT through an ANCOVA analysis taking MAT as the predictor variable, *T*_min_, *T*_opt_, or *T*_max_, as the response variable, and sampling season as a covariate. We also used a *t* test to identify if sampling season had a significant effect on the temperature relationships of a subset of 10 soils (Table S2) that were sampled in both winter and summer. Additionally, the temperature relationship indices were regressed against the other environmental and soil properties measured (MAP, pH, and SOM). To evaluate if C availability and temperature were interrelated in our survey, we estimated C availability as the rate of microbial C use per SOM and correlated it with MAT (see File S2 for details). Finally, to confirm the predictors of the temperature relationship indices, we used a multiple regression model. Using the *lm* function, we modeled the temperature relationship indices as a function of the different environmental factors and soil properties according to the regression equation 4.

(4) $$y=a\;+\;b_{\text{pH}}\;\text{*pH}\;+\;b_{\text{SOM}}\;\text{*SOM}\;+\;b_{\text{MAT}}\;\text{*MAT}\;+\;b_{\text{MAP}}\;\text{*MAP}$$

where *y* is one of the temperature relationship indices (*T*_min_, *T*_opt_, or *T*_max_) as a dependent variable, *a* is the intercept, and *b*_pH_, *b*_SOM_, *b*_MAT_, and *b*_MAP_ are the regression coefficients of pH, SOM, MAT, and MAP, respectively. The data were checked for multicollinearity using the variance inflation factors before analyses.

With the ASVs obtained from the microbial community analyses, we calculated diversity metrics for bacteria and fungi. We calculated the Shannon index (α-diversity) and richness for bacterial and fungal communities in each site before filtering and transformation to even sampling depth. Moreover, in the β-diversity analysis, ASVs were filtered by keeping only ASVs with at least 5 counts, and samples were then transformed to even sampling depth with the function transform_sample_counts from the phyloseq package (95). We calculated the Bray-Curtis dissimilarity (β-diversity) matrix and visualized the differences in the bacterial and fungal communities across sites with a nonmetric multidimensional scaling (NMDS) ordination. To interlink microbial diversity with temperature trait distributions, we regressed the α-diversity and richness against bacterial and fungal *T*_min_ values. Additionally, environmental parameters and soil properties were also regressed to bacterial and fungal α-diversity and richness. Further, bacterial or fungal growth *T*_min_ values, environmental parameters, and soil properties were fitted onto the ordination space (Bray-Curtis NMDS) to assess correlations between these variables and the bacterial and fungal community composition using the function envfit from the vegan package (96). The significance of fitted vectors was assessed using permutation of the variables (96). Finally, we correlated the ASVs’ relative abundance with *T*_min_ values to identify warm- and cold-adapted taxa (see File S2 for details). All statistical analyses were done in R version 4.0.3 (83).

### Data availability.

The sequencing data obtained have been deposited in European Nucleotide Archive (ENA) with the primary accession number PRJEB45259. The raw data for bacterial growth, fungal growth, and respiration rate have been deposited in figshare under doi 10.6084/m9.figshare.21967388.
