# Supplementary material for: Variation in Temperature Dependences across Europe Reveals the Climate Sensitivity of Soil Microbial Decomposers
Source: Appl Environ Microbiol. 2023 May 10;89(5):e02090-22. doi: 10.1128/aem.02090-22 (PMC10231190; doi:10.1128/aem.02090-22)
Supplement: Supplemental file 2 — Supplemental material. Download aem.02090-22-s0002.docx, DOCX file, 1.7 MB [file aem.02090-22-s0002.docx]

**Supplementary Information for**

**Variation in soil microbial temperature dependences across Europe reveals the climate-sensitivity of decomposer temperature trait distributions**

**Running title**: Climate adapted decomposers

Carla Cruz-Paredes^a*^, Dániel Tájmel^a^ and Johannes Rousk^a^

^a^Microbial Ecology, Department of Biology, Lund University, Lund, Sweden 22362

*corresponding author

**E-mail**: carla.cruz_paredes@biol.lu.se

**This PDF file includes:**

Supplementary methods

Figures S1 to S3

Tables S1 to S8

**Supplementary methods**

*Microbial temperature dependences*

Subsamples of the soils were transferred to vials that were exposed to ten different temperatures from 0°C to 45°C in 5°C intervals in water baths, as shown in the diagram below:

The exposure to different temperatures during the incubation step was adapted for the different temperatures as follows:

| Incubation temperature | Bacterial growth | Fungal growth | Respiration rate |
| --- | --- | --- | --- |
| 0°C | 32 h | 64 h | 149 h |
| 5°C | 16 h | 32 h | 80 h |
| 10°C | 8 h | 16 h | 48 h |
| 15°C | 4 h | 8 h | 30 h |
| 20°C | 2 h | 4 h | 18 h |
| 25°C | 2 h | 4 h | 18 h |
| 30°C | 1 h | 2 h | 6 h |
| 35°C | 1 h | 2 h | 3 h |
| 40°C | 1 h | 2 h | 3 h |
| 45°C | 1 h | 2 h | 3 h |

These times ensure a similar level of C use in all treatments, within these time periods no change in growth or respiration rates due to altered conditions occurs, except for the direct temperature effect on rates.

*Bacterial growth*

The bacterial growth was estimated using leucine incorporation in bacteria extracted from soil using the homogenization/centrifugation technique (Bååth, Pettersson, & Söderberg, 2001) with modifications. A bacterial suspension was extracted from 0.5 g of soil mixed with 20 ml distilled water. The incorporation of radioactive leucine was done by incubating 1.5 ml of the bacterial suspension with 2 μl radioactively labelled leucine, ([^3^H]Leu, 185 MBq ml^-1^, 2 TBq mmol^-1^, Perkin Elmer) combined with non-labelled leucine to a resulting concentration of 280 nM in the sample. After the incubation time growth was terminated by addition of trichlorinated acetic acid. Samples were then washed according to Bååth, Pettersson, & Söderberg (2001).

*Fungal growth*

Fungal growth was estimated using the technique of acetate incorporation into ergosterol (Bååth, 2001; Rousk, Brookes, & Bååth, 2009). Briefly, 0.3 g of soil were mixed with 1.95 ml of water, 20 μl of 1-([^14^C] acetic acid (sodium salt, 37 MBq mL^-1^, 2.10 GBq mmol^-1^, Perkin Elmer) combined with 30 μl 16 mM non-labelled sodium acetate, yielding a final concentration of ca. 220 μM in the sample. After the incubation time growth was terminated by the addition of formalin. Ergosterol was then extracted, separated, and quantified using high-performance liquid chromatography and a UV detector and an external standard (Rousk, Brookes, & Bååth, 2009). The ergosterol peak was collected in a fraction collector and the incorporated acetate was measured using liquid scintillation.

*C availability*

As a proxy for C availability the microbial growth and respiration measurements in C units were added and divided by the SOM content in each soil to capture the resource quality experienced by the in situ microbial community within the studied soil samples. For bacterial growth, the ^3^H-leucine incorporation values were first converted to thymidine incorporation with the relationship that 10 pmol of leucine are equivalent to 1 pmol of thymidine (Tang et al., 2022). Further, the rate of thymidine incorporation was converted to units of C in bacterial biomass using the conversion factor in Soares and Rousk (2019). For fungal growth the amount of incorporated acetate into extracted ergosterol was expressed in units of C using the conversion factor in Soares and Rousk (2019).

*Seasonal variation and microbial growth envelope*

To test if the annual amplitudes in environmental temperature variation could explain microbial adaptation, the difference between the mean temperature of the coldest month and mean temperature of the warmest month (ten years means 2009-2018) was estimated. The bacterial and fungal growth envelopes, or width of the temperature relationships were calculated as the differences between the ultimate endpoints of the modelled temperature relationships, i.e., by substracting *T_max_*-*T_min_*. The microbial growth envelope was then regressed against the amplitude to understand if microbial communities were adapted to stronger variations in temperature.

*Microbial community*

Extracted DNA was sent to BGI Tech Solutions (Hong Kong) for amplicon sequencing according to their standard protocol (as provided by BGI Tech Solutions). Two methods were used to test the whole genomics DNA samples in BGI: 1) Fluorescence quantitative and 2) agarose gel electrophoresis. Concentration was tested by fluorescence or microplate Reader (e.g., Qubit Fluorometer and Qubit dsDNA BR Assay Kit). Sample integrity was tested by agarose gel electrophoresis (concentration of agarose gel:1%, voltage:150 V, electrophoresis time:40 min). The qualified samples further entered the library preparation process as follows, 30ng DNA sample and fusion primer were used to configure PCR reaction system. PCR reaction parameters were set as standard for PCR amplification. Agencourt AMPure XP beads were then used to purify DNA and dissolve in elution buffer. After proper labelling, construction of libraries was finished. Library was qualified by the Agilent Technologies 2100 bioanalyzer. The qualified libraries were sequenced pair-end on the Hiseq 2500.

The relative abundance of the annotated ASVs was correlated with bacterial and fungal *T_min_* values to identify warm- and cold-adapted taxa. The ten ASVs most correlated with *T_min_* (positively and negatively) were further investigated. These ASVs were entered in databases to identify if they have been reported before in specific locations on climates. Bacterial ASVs were searched in the database Microbe Atlas (Rodrigues et al., 2017). From this database the soil habitat with the highest number of sequencing reads mapping to each ASVs was reported (Table S7). Fungal ASVs were explored in the database Global Fungi (Vetrovsky et al., 2020). From this database the MAT range where each ASV was more frequently identified was reported (Table S7).

| **Table S1.** Information for the 72 soils used. Location, latitude and longitude coordinates, month and year of sampling are reported. Mean annual temperature (MAT), mean annual precipitation (MAP), soil pH, electrical conductivity (EC), and soil organic matter (SOM) and *R*^2^-values for microbial growth and respiration rate temperature dependences for each soil are reported. | | | | | | | | | | | |
| --- | --- | --- | --- | --- | --- | --- | --- | --- | --- | --- | --- |
| **Location** | **Latitude** | **Longitude** | **Sampling date** | **MAT (°C)** | **MAP (mm)** | **pH** | **EC**  **(µS cm^-1^)** | **SOM (%)** | **Bacterial growth *R^2^*** | **Fungal growth *R^2^*** | **Respiration rate *R^2^*** |
| Gibraltar, ES | 36.12 | -5.34 | Jun-2019 | 18.3 | 645 | 7.0 | 65.8 | 11.9 | 0.99 | 0.97 | 0.96 |
| El Torcal National Park, ES | 36.95 | -4.56 | Jun-2019 | 17.4 | 568 | 7.3 | 67.2 | 18.4 | 0.98 | 0.99 | 0.98 |
| Granada, ES | 37.19 | -3.65 | Jun-2019 | 15.7 | 457 | 7.1 | 105.3 | 6.7 | 0.99 | 0.91 | 0.98 |
| Malaga, ES | 36.58 | -4.59 | Jun-2019 | 17.4 | 568 | 7.3 | 128.9 | 9.4 | 0.99 | 0.95 | 0.87 |
| Malaga, ES | 36.91 | -4.81 | Jun-2019 | 17.4 | 568 | 7.5 | 67.9 | 8.2 | 0.99 | 0.86 | 0.99 |
| Abisko, SE | 68.32 | 18.68 | Jun-2019 | -3.1 | 815 | 5.3 | 19.9 | 62.1 | 0.99 | 0.99 | 0.99 |
| Dartmoor, UK | 50.59 | -3.77 | Jul-2019 | 10.2 | 1335 | 6.8 | 25.1 | 24.2 | 0.98 | 0.99 | 0.97 |
| Stonehenge, UK | 51.18 | -1.83 | Jul-2019 | 10.3 | 765 | 7.0 | 76.6 | 20.7 | 0.99 | 0.97 | 0.99 |
| Cornwall, UK | 50.08 | -5.54 | Jul-2019 | 11.3 | 1267 | 6.9 | 103.4 | 8.9 | 0.98 | 0.95 | 0.98 |
| Snowdon, UK | 53.08 | -4.05 | Jul-2019 | 10.3 | 1264 | 7.6 | 21.0 | 10.2 | 0.99 | 0.96 | 0.97 |
| Tintagel, UK | 50.67 | -4.76 | Jul-2019 | 11.3 | 1267 | 6.1 | 369.0 | 38.1 | 0.98 | 0.98 | 0.99 |
| Braies, IT | 46.69 | 12.08 | Jul-2019 | 0.9 | 1533 | 6.4 | 67.3 | 44.9 | 0.99 | 0.96 | 0.97 |
| Sarentino, IT | 46.70 | 11.29 | Jul-2019 | 3.7 | 1384 | 6.6 | 29.1 | 19.0 | 0.89 | 0.97 | 0.94 |
| Avelengo, IT | 46.68 | 11.26 | Jul-2019 | 3.7 | 1384 | 6.1 | 30.4 | 11.6 | 0.99 | 0.99 | 0.95 |
| Rome, IT | 41.89 | 12.49 | Jul-2019 | 16.8 | 869 | 7.0 | 219.0 | 18.5 | 0.99 | 0.96 | 0.99 |
| Tivoli, IT | 41.96 | 12.80 | Jul-2019 | 16.1 | 869 | 7.3 | 129.2 | 27.3 | 0.99 | 0.99 | 0.99 |
| Pisa, IT | 43.72 | 10.32 | Jul-2019 | 15.0 | 815 | 7.0 | 28.9 | 10.5 | NA | 0.99 | 0.98 |
| Chianni, IT | 43.48 | 10.65 | Jul-2019 | 14.5 | 725 | 7.0 | 103.6 | 16.7 | 0.99 | 0.90 | 0.98 |
| Terracina, IT | 41.28 | 13.14 | Jul-2019 | 16.5 | 825 | 7.6 | 55.0 | 6.8 | 0.99 | 0.98 | 0.99 |
| Barrea, IT | 41.78 | 13.94 | Jul-2019 | 15.6 | 840 | 7.5 | 49.7 | 30.1 | 0.98 | 0.98 | 0.98 |
| Sabaudia, IT | 41.29 | 13.05 | Jul-2019 | 16.5 | 825 | 6.8 | 49.8 | 18.8 | 0.98 | 0.97 | 0.98 |
| Navarra, ES | 42.80 | -2.14 | Aug-2019 | 11.1 | 955 | 6.6 | 202.0 | 19.0 | 0.96 | 0.99 | 0.98 |
| Bergara, ES | 43.12 | -2.40 | Aug-2019 | 14.1 | 1078 | 6.8 | 95.9 | 28.6 | 0.99 | 0.97 | 0.99 |
| Rennertehausen, DE | 51.02 | 8.69 | Aug-2019 | 9.1 | 792 | 5.9 | 74.3 | 12.8 | 0.99 | 0.95 | 0.99 |
| Rennertehausen, DE | 51.02 | 8.69 | Aug-2019 | 9.1 | 792 | 6.4 | 45.2 | 5.8 | 0.99 | 0.98 | 0.97 |
| Budapest, HU | 47.39 | 19.05 | Aug-2019 | 11.7 | 566 | 7.7 | 39.0 | 4.9 | 0.99 | 0.97 | 0.99 |
| London, UK | 51.58 | -0.13 | Aug-2019 | 10.7 | 689 | 8.0 | 25.5 | 11.8 | 0.99 | 0.99 | 0.96 |
| Varjisån, SE | 66.02 | 19.85 | Aug-2019 | -0.7 | 561 | 3.9 | 22.2 | 93.7 | 0.97 | 0.98 | 0.98 |
| Varjisån, SE | 66.02 | 19.86 | Aug-2019 | -0.7 | 561 | 4.4 | 24.9 | 26.5 | 0.99 | 0.98 | 0.99 |
| Kryddgrovan, SE | 64.98 | 19.56 | Aug-2019 | 1.9 | 539 | 4.4 | 19.8 | 74.5 | 0.97 | 0.99 | 0.98 |
| Kryddgrovan, SE | 64.98 | 19.57 | Aug-2019 | 1.9 | 539 | 4.8 | 32.8 | 40.9 | 0.99 | 0.96 | 0.99 |
| Akkelis, SE | 66.12 | 17.66 | Aug-2019 | -0.8 | 511 | 5.2 | 11.8 | 41.7 | 0.98 | 0.98 | 0.99 |
| Biatorbagy, HU | 47.48 | 18.81 | Dec-2019 | 11.7 | 587 | 7.9 | 65.7 | 8.3 | 0.97 | 0.99 | 0.98 |
| Reimersholme, SE | 59.32 | 18.02 | Dec-2019 | 7.2 | 459 | 6.5 | 19.1 | 11.1 | 0.95 | 0.97 | 0.99 |
| Elgholmen, SE | 59.01 | 17.01 | Dec-2019 | 6.8 | 518 | 6.5 | 14.8 | 15.6 | 0.98 | 0.95 | 0.98 |
| Björnlunda, SE | 59.06 | 17.16 | Dec-2019 | 6.8 | 518 | 6.4 | 16.7 | 12.4 | 0.99 | 0.99 | 0.99 |
| Vaggeryd, SE | 57.52 | 14.13 | Dec-2019 | 6.5 | 811 | 6.3 | 23.6 | 25.7 | 0.99 | 0.99 | 0.99 |
| Alingsås, SE | 57.94 | 12.55 | Dec-2019 | 7.4 | 864 | 5.5 | 14.1 | 10.0 | 0.99 | 0.99 | 0.98 |
| Alingsås, SE | 57.94 | 12.55 | Dec-2019 | 7.4 | 864 | 7.4 | 16.6 | 3.6 | 0.99 | 0.99 | 0.99 |
| Alingsås, SE | 57.94 | 12.55 | Dec-2019 | 7.4 | 864 | 5.7 | 11.6 | 9.8 | 0.99 | 0.99 | 0.97 |
| St. John Abadesses, ES | 42.23 | 2.29 | Jan-2020 | 9.5 | 828 | 6.8 | 50.2 | 8.8 | 0.99 | 0.98 | 0.99 |
| Hrase, SI | 46.17 | 14.44 | Jan-2020 | 9.5 | 1641 | 7.0 | 39.0 | 11.1 | 0.98 | 0.90 | 0.97 |
| Hrase, SI | 46.18 | 14.44 | Jan-2020 | 9.5 | 1641 | 3.9 | 31.9 | 49.6 | 0.99 | 0.95 | 0.98 |
| Stuttgart, DE | 48.96 | 9.18 | Jan-2020 | 10.6 | 684 | 6.3 | 183.9 | 6.8 | 0.99 | 0.99 | 0.97 |
| Rennertehausen, DE | 51.02 | 8.69 | Jan-2020 | 9.1 | 792 | 6.7 | 54.2 | 5.4 | 0.99 | 0.98 | 0.97 |
| Odense, DK | 55.37 | 10.50 | Jan-2020 | 9.2 | 643 | 6.7 | 50.8 | 8.5 | 0.99 | 0.98 | 0.99 |
| Hatzfeld, DE | 51.00 | 8.54 | Jan-2020 | 9.1 | 792 | 6.6 | 21.3 | 16.9 | 0.99 | 0.97 | 0.98 |
| Vercelli, IT | 45.40 | 8.20 | Jan-2020 | 13.7 | 792 | 6.4 | 14.5 | 8.5 | 0.99 | 0.78 | 0.98 |
| Vercelli, IT | 45.40 | 8.20 | Jan-2020 | 13.7 | 792 | 6.8 | 25.8 | 6.5 | 0.99 | 0.97 | 0.98 |
| Szekesfehervar, HU | 47.19 | 18.41 | Jan-2020 | 11.5 | 608 | 7.0 | 80.4 | 5.4 | 0.97 | 0.95 | 0.99 |
| Kew, UK | 51.48 | -0.30 | Jan-2020 | 10.4 | 773 | 7.2 | 37.2 | 34.1 | 0.99 | 0.98 | 0.97 |
| Kew, UK | 51.48 | -0.30 | Jan-2020 | 10.4 | 773 | 6.0 | 51.4 | 45.7 | 0.98 | 0.96 | 0.95 |
| Bergara, ES | 43.12 | -2.43 | Jan-2020 | 14.1 | 1174 | 6.2 | 50.2 | 12.6 | 0.99 | 0.99 | 0.99 |
| Terracina, IT | 41.28 | 13.14 | Jan-2020 | 16.5 | 825 | 6.7 | 21.3 | 3.6 | 0.99 | 0.99 | 0.96 |
| London, UK | 51.43 | -0.04 | Jan-2020 | 10.4 | 773 | 6.6 | 31.5 | 14.0 | 0.99 | 0.97 | 0.98 |
| Bergara, ES | 43.12 | -2.43 | Jan-2020 | 14.1 | 1174 | 6.5 | 23.6 | 15.8 | 0.98 | 0.99 | 0.98 |
| Lund, SE | 55.71 | 13.21 | Feb-2020 | 8.7 | 667 | 6.7 | 22.3 | 8.6 | 0.99 | 0.97 | 0.96 |
| Sabaudia, IT | 41.29 | 13.05 | Feb-2020 | 16.5 | 825 | 6.5 | 15.0 | 3.6 | 0.99 | 0.99 | 0.98 |
| Santiago de Compostela, ES | 42.83 | -8.47 | Feb-2020 | 13.0 | 1536 | 6.2 | 38.1 | 11.4 | 0.99 | 0.96 | 0.99 |
| Santiago de Compostela, ES | 42.83 | -8.47 | Feb-2020 | 13.0 | 1536 | 4.6 | 39.4 | 52.8 | 0.98 | 0.97 | 0.98 |
| Helsinki, FI | 60.19 | 24.94 | Feb-2020 | 5.9 | 590 | 6.5 | 28.9 | 9.5 | 0.99 | 0.97 | 0.99 |
| Helsinki, FI | 60.19 | 24.94 | Feb-2020 | 5.9 | 590 | 6.5 | 30.4 | 22.1 | 0.99 | 0.99 | 0.99 |
| Helsinki, FI | 60.19 | 24.94 | Feb-2020 | 5.9 | 590 | 6.3 | 35.7 | 36.5 | 0.98 | 0.93 | 0.99 |
| Helsinki, FI | 60.19 | 24.94 | Feb-2020 | 5.9 | 590 | 6.3 | 23.9 | 56.4 | 0.99 | 0.94 | 0.99 |
| Athens, GR | 37.97 | 23.80 | Feb-2020 | 17.6 | 579 | 6.4 | 181.4 | 16.3 | 0.97 | 0.98 | 0.96 |
| Athens, GR | 37.97 | 23.80 | Feb-2020 | 17.6 | 579 | 6.8 | 90.2 | 3.5 | 0.99 | 0.94 | 0.97 |
| t Harde, NL | 52.40 | 5.91 | Feb-2020 | 10.5 | 802 | 4.5 | 111.3 | 73.4 | 0.98 | 0.97 | 0.97 |
| t Harde, NL | 52.40 | 5.91 | Feb-2020 | 10.5 | 802 | 3.9 | 82.6 | 78.0 | 0.99 | 0.98 | 0.97 |
| Lund, SE | 55.69 | 13.40 | Feb-2020 | 8.7 | 667 | 4.9 | 93.7 | 21.3 | 0.99 | 0.96 | 0.96 |
| Höör, SE | 55.96 | 13.53 | Feb-2020 | 8.7 | 667 | 4.4 | 50.6 | 92.3 | 0.98 | 0.92 | 0.98 |
| Lund, SE | 55.69 | 13.40 | Feb-2020 | 8.7 | 667 | 6.0 | 49.9 | 9.3 | 0.98 | 0.94 | 0.99 |
| Abisko, SE | 68.32 | 18.68 | Jun-2020 | -3.1 | 815 | 4.9 | 145.5 | 64.4 | 0.99 | 0.99 | 0.99 |

**Figure S1.** Minimum temperature (*T_min_*) for **A)** bacterial growth **B)** fungal growth and **C)** respiration rate, optimum temperature (*T_opt_*) for **D)** bacterial growth, and **E)** fungal growth, and maximum temperature (*T_max_*) for **F)** bacterial growth, and **G)** fungal growth regressed against mean annual temperature (MAT) across the European gradient. Colors represent sampling time. ANCOVA results are reported for mean annual temperature (M), season (S) and the interaction (M:S).

**M** *F_1,67_*= 22.1, *p*= 0.00001

**S** *F_1,67_*= 0.2, *p*= 0.67

**M:S** *F_1,67_*= 1.8, *p*= 0.18

**M** *F_1,68_*= 4.1, *p*= 0.04

**S** *F_1,68_*= 5.2, *p*= 0.03

**M:S** *F_1,68_*= 0.2, *p*=0.67

**M** *F_1,68_*= 21.8, *p*= 0.00001

**S** *F_1,68_*= 0.3, *p*= 0.60

**M:S** *F_1,68_*= 0.0, *p*=0.83

**M** *F_1,67_*= 15.2, *p*= 0.0002

**S** *F_1,67_*= 0.1, *p*= 0.80

**M:S** *F_1,67_*= 0.1, *p*=0.72

**M** *F_1,67_*= 4.5, *p*=0.04

**S** *F_1,67_*= 0.0, *p*= 0.91

**M:S** *F_1,67_*= 1.2, *p*=0.28

**M** *F_1,68_*= 11.5, *p*= 0.001

**S** *F_1,68_*= 0.1, *p*= 0.82

**M:S** *F_1,68_*= 0.6, *p*= 0.45

**M** *F_1,68_*= 0.1, *p*= 0.71

**S** *F_1,68_*= 0.0, *p*= 0.99

**M:S** *F_1,68_*= 0.0, *p*=0.91

**Table S2.** Selected soils originated from nearby sites that were sampled both in winter and summer.

| **Location** | **Latitude** | **Longitude** | **Sampling date** |
| --- | --- | --- | --- |
| Bergara, ES | 43.12 | -2.40 | Aug-2019 |
| Bergara, ES | 43.12 | -2.43 | Jan-2020 |
| London, UK | 51.58 | -0.13 | Aug-2019 |
| London, UK | 51.43 | -0.04 | Jan-2020 |
| Rennertehausen, DE | 51.02 | 8.69 | Aug-2019 |
| Rennertehausen, DE | 51.02 | 8.69 | Jan-2020 |
| Sabaudia, IT | 41.29 | 13.05 | Jul-2019 |
| Sabaudia, IT | 41.29 | 13.05 | Feb-2020 |
| Terracina, IT | 41.28 | 13.14 | Jul-2019 |
| Terracina, IT | 41.28 | 13.14 | Jan-2020 |


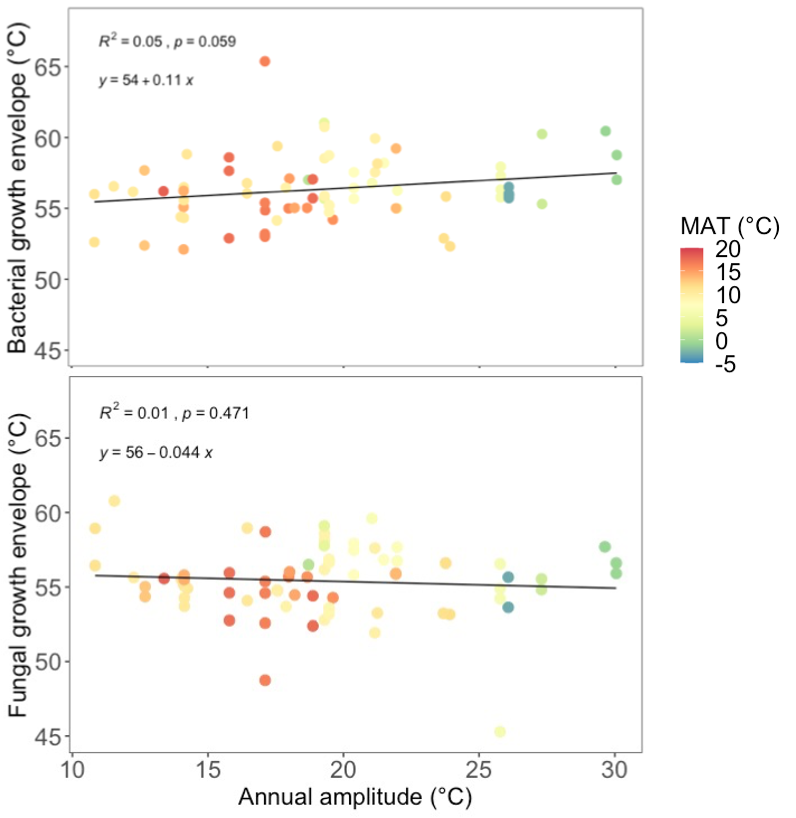


**Figure S2**. Annual amplitude in environmental temperature variation (difference between the warmest month (summer) and the coldest month (winter) temperatures) regressed against the width of the temperature relationships (growth envelope = *T_max_* – *T_min_*) for bacterial and fungal growth. Colors represent MAT across the sampled gradient.

**Table S3**. Regressions of minimum temperature (*T_min_*), optimum temperature (*T_opt_*) and maximum temperature (*T_max_*) for microbial growth (bacterial, fungal) and respiration with mean annual temperature (MAT), the average temperature in the warmest month (summer) and the coldest month (winter). Significant regressions are in bold.

|  | **Equation** | ***F*** | ***df*** | ***R^2^*** | ***p*** |
| --- | --- | --- | --- | --- | --- |
| **Bacterial *T_min_*** |  |  |  |  |  |
| MAT | *y*= -11 + 0.20*x* | 22.1 | 1, 69 | 0.24 | **<0.001** |
| Summer | *y*= -13 + 0.22*x* | 17.3 | 1, 69 | 0.20 | **<0.001** |
| Winter | *y*= -9 + 0.15*x* | 20.6 | 1, 69 | 0.23 | **<0.001** |
| **Fungal *T_min_*** |  |  |  |  |  |
| MAT | *y*= -8.3 + 0.07*x* | 3.9 | 1, 70 | 0.05 | 0.052 |
| Summer | *y*= -9.6 + 0.10*x* | 5.7 | 1, 70 | 0.08 | **0.020** |
| Winter | *y*= -7.6 + 0.04*x* | 2.4 | 1, 70 | 0.03 | 0.128 |
| **Respiration *T_min_*** |  |  |  |  |  |
| MAT | *y*= -6.5 + 0.10*x* | 22.3 | 1, 70 | 0.24 | **<0.001** |
| Summer | *y*= -7.5 + 0.10*x* | 14.3 | 1, 70 | 0.17 | **<0.001** |
| Winter | *y*= -5.6 + 0.08*x* | 23.1 | 1, 70 | 0.25 | **<0.001** |
| **Bacterial *T_opt_*** |  |  |  |  |  |
| MAT | *y*= 32 + 0.10*x* | 15.6 | 1, 69 | 0.18 | **<0.001** |
| Summer | *y*= 30 + 0.14*x* | 21.9 | 1, 69 | 0.24 | **<0.001** |
| Winter | *y*= 33 + 0.06*x* | 9.7 | 1, 69 | 0.12 | **0.003** |
| **Fungal *T_opt_*** |  |  |  |  |  |
| MAT | *y*= 33 + 0.19*x* | 11.8 | 1, 70 | 0.14 | **<0.001** |
| Summer | *y*= 30 + 0.26*x* | 16.4 | 1, 70 | 0.19 | **<0.001** |
| Winter | *y*= 35 + 0.12*x* | 8.3 | 1, 70 | 0.11 | **0.005** |
| **Bacterial *T_max_*** |  |  |  |  |  |
| MAT | *y*= 47 + 0.05*x* | 5.5 | 1, 69 | 0.07 | **0.022** |
| Summer | *y*= 46 + 0.06*x* | 5.5 | 1, 69 | 0.07 | **0.022** |
| Winter | *y*= 47 + 0.03*x* | 4.6 | 1, 69 | 0.06 | **0.036** |
| **Fungal *T_max_*** |  |  |  |  |  |
| MAT | *y*= 48 - 0.01*x* | 0.1 | 1, 70 | 0.00 | 0.703 |
| Summer | *y*= 49 - 0.05*x* | 1.4 | 1, 70 | 0.02 | 0.237 |
| Winter | *y*= 48 + 0.00*x* | 0.0 | 1, 70 | 0.00 | 0.928 |

**Table S4**. Regressions of minimum temperature (*T_min_*), optimum temperature (*T_opt_*) and maximum temperature (*T_max_*) for microbial growth (bacterial, fungal) and respiration with soil and environmental properties: mean annual precipitation (MAP), pH and soil organic matter (SOM). Significant regressions are in bold.

|  | **Equation** | ***F*** | ***df*** | ***R^2^*** | ***p*** |
| --- | --- | --- | --- | --- | --- |
| **Bacterial *T_min_*** |  |  |  |  |  |
| MAP | y = -9.2 + 0.00x | 0.2 | 1, 69 | 0.00 | 0.671 |
| pH | y = -13.0 + 0.66x | 7.1 | 1, 69 | 0.09 | **0.009** |
| SOM | y = -8.4 - 0.02x | 3.3 | 1, 69 | 0.05 | 0.073 |
| **Fungal *T_min_*** |  |  |  |  |  |
| MAP | y = -6.9 - 0.00x | 1.6 | 1, 70 | 0.02 | 0.210 |
| pH | y = -10.7 + 0.49x | 6.8 | 1, 70 | 0.09 | **0.011** |
| SOM | y = -7.2 - 0.02x | 4.2 | 1, 70 | 0.06 | **0.044** |
| **Respiration *T_min_*** |  |  |  |  |  |
| MAP | y = -5.5 + 0.00x | 0.0 | 1, 70 | 0.00 | 0.967 |
| pH | y = -8.2 + 0.42x | 11.9 | 1, 70 | 0.15 | **0.001** |
| SOM | y = -5.2 - 0.01x | 4.4 | 1, 70 | 0.06 | **0.041** |
| **Bacterial *T_opt_*** |  |  |  |  |  |
| MAP | *y*= 33 + 0.00*x* | 0.0 | 1, 69 | 0.00 | 0.973 |
| pH | *y*= 30 + 0.39*x* | 7.6 | 1, 69 | 0.10 | **0.008** |
| SOM | *y*= 33 - 0.02*x* | 15.1 | 1, 69 | 0.18 | **<0.001** |
| **Fungal *T_opt_*** |  |  |  |  |  |
| MAP | *y*= 36 - 0.00*x* | 1.8 | 1, 70 | 0.02 | 0.185 |
| pH | *y*= 27 + 1.32*x* | 24.5 | 1, 70 | 0.26 | **<0.001** |
| SOM | *y*= 36 - 0.06*x* | 22.8 | 1, 70 | 0.25 | **<0.001** |
| **Bacterial *T_max_*** |  |  |  |  |  |
| MAP | *y*= 47 + 0.00*x* | 1.4 | 1, 69 | 0.02 | 0.245 |
| pH | *y*= 46 + 0.22*x* | 4.3 | 1, 69 | 0.06 | **0.042** |
| SOM | *y*= 48 - 0.01*x* | 5.6 | 1, 69 | 0.07 | **0.021** |
| **Fungal *T_max_*** |  |  |  |  |  |
| MAP | *y*= 47 + 0.00*x* | 2.0 | 1, 70 | 0.03 | 0.158 |
| pH | *y*= 47 + 0.14*x* | 0.6 | 1, 70 | 0.01 | 0.429 |
| SOM | *y*= 48 - 0.01*x* | 0.9 | 1, 70 | 0.01 | 0.351 |

**Table S5**. Multiple regression analysis of minimum temperature (*T_min_*), optimum temperature (*T_opt_*) and maximum temperature (*T_max_*), for microbial growth (bacterial, fungal) and respiration with soil and environmental properties: pH, soil organic matter (SOM), mean annual temperature (MAT) and mean annual precipitation (MAP). Significant values are in bold.

| **Bacterial *T_min_*** | Estimate | Std. Error | *t* | *p* | *F* (model) | *Df* (model) | *R^2^* (model) | p (model) |
| --- | --- | --- | --- | --- | --- | --- | --- | --- |
| (Intercept) | -1.31e+01 | 2.57e+00 | -5.09 | **<0.001** | 5.5 | 4, 66 | 0.25 | **<0.001** |
| pH | 2.76e-01 | 3.60e-01 | 0.77 | 0.45 |  |  |  |  |
| SOM | 9.93e-03 | 1.54e-02 | 0.64 | 0.52 |  |  |  |  |
| MAT | 1.93e-01 | 5.27e-02 | 3.66 | **<0.001** |  |  |  |  |
| MAP | 3.33e-04 | 7.57e-04 | 0.44 | 0.66 |  |  |  |  |
| **Bacterial *T_opt_*** |  |  |  |  |  |  |  |  |
| (Intercept) | 3.35e+01 | 1.49e+00 | 22.50 | **<0.001** | 5.3 | 4, 66 | 0.24 | **<0.001** |
| pH | -1.58e-01 | 2.08e-01 | -0.76 | 0.45 |  |  |  |  |
| SOM | -2.08e-02 | 8.94e-03 | -2.33 | **0.02** |  |  |  |  |
| MAT | 7.27e-02 | 3.05e-02 | 2.38 | **0.02** |  |  |  |  |
| MAP | -4.02e-05 | 4.38e-04 | -0.09 | 0.93 |  |  |  |  |
| **Bacterial *T_max_*** |  |  |  |  |  |  |  |  |
| (Intercept) | 4.66e+01 | 1.20e+00 | 38.82 | **<0.001** | 2.1 | 4, 66 | 0.11 | 0.08 |
| pH | 4.05e-02 | 1.68e-01 | 0.24 | 0.81 |  |  |  |  |
| SOM | -7.47e-03 | 7.22e-03 | -1.04 | 0.31 |  |  |  |  |
| MAT | 2.43e-02 | 2.47e-02 | 0.99 | 0.33 |  |  |  |  |
| MAP | 4.38e-04 | 3.54e-04 | 1.24 | 0.22 |  |  |  |  |
| **Fungal *T_min_*** |  |  |  |  |  |  |  |  |
| (Intercept) | -9.41e+00 | 2.14e+00 | -4.40 | **<0.001** | 2.2 | 4, 67 | 0.11 | 0.08 |
| pH | 3.44e-01 | 3.00e-01 | 1.15 | 0.25 |  |  |  |  |
| SOM | -2.47e-03 | 1.28e-02 | -0.19 | 0.85 |  |  |  |  |
| MAT | 3.26e-02 | 4.35e-02 | 0.75 | 0.46 |  |  |  |  |
| MAP | -7.52e-04 | 6.30e-04 | -1.19 | 0.24 |  |  |  |  |
| **Fungal *T_opt_*** |  |  |  |  |  |  |  |  |
| (Intercept) | 3.19e+01 | 2.93e+00 | 10.89 | **<0.001** | 7.9 | 4, 67 | 0.32 | **<0.001** |
| pH | 6.36e-01 | 4.11e-01 | 1.55 | 0.13 |  |  |  |  |
| SOM | -2.93e-02 | 1.76e-02 | -1.66 | 0.10 |  |  |  |  |
| MAT | 6.39e-02 | 5.96e-02 | 1.07 | 0.29 |  |  |  |  |
| MAP | -1.19e-03 | 8.65e-04 | -1.37 | 0.17 |  |  |  |  |
| **Fungal *T_max_*** |  |  |  |  |  |  |  |  |
| (Intercept) | 4.66e+01 | 1.98e+00 | 23.53 | **<0.001** | 1.1 | 4, 67 | 0.06 | 0.37 |
| pH | 1.74e-01 | 2.78e-01 | 0.63 | 0.53 |  |  |  |  |
| SOM | -7.23e-03 | 1.19e-02 | -0.61 | 0.55 |  |  |  |  |
| MAT | -4.62e-02 | 4.03e-02 | -1.15 | 0.26 |  |  |  |  |
| MAP | 8.90e-04 | 5.84e-04 | 1.52 | 0.13 |  |  |  |  |
| **Respiration *T_min_*** |  |  |  |  |  |  |  |  |
| (Intercept) | -8.42e+00 | 1.31e+00 | -6.45 | **<0.001** | 6.2 | 4, 67 | 0.27 | **<0.001** |
| pH | 2.93e-01 | 1.83e-01 | 1.60 | 0.11 |  |  |  |  |
| SOM | 7.56e-03 | 7.84e-03 | 0.96 | 0.34 |  |  |  |  |
| MAT | 8.84e-02 | 2.65e-02 | 3.33 | **0.001** |  |  |  |  |
| MAP | -1.84e-06 | 3.85e-04 | -0.005 | 0.99 |  |  |  |  |

**
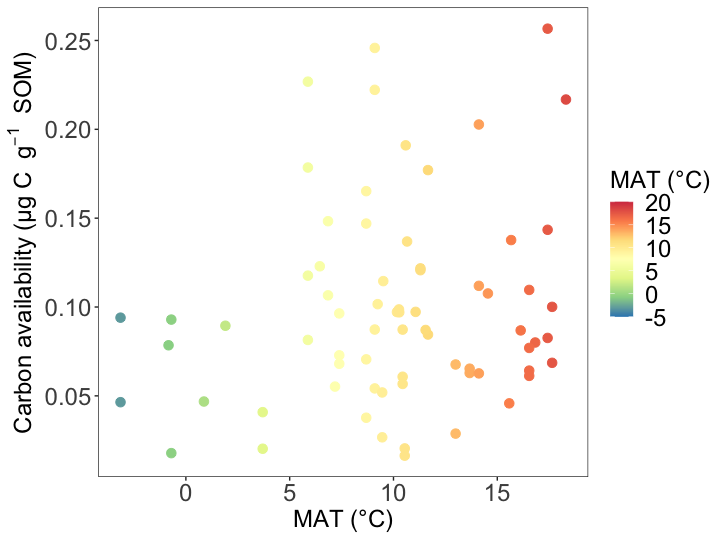
Figures S3.** Correlation between C availability estimated as the rate of microbial C-use per soil organic matter (SOM) and mean annual temperature (MAT). *Pearson’s corr*= 0.18 *p*= 0.16. Colors represent MAT across the sampled gradient.

**Table S6.** Regressions of bacterial and fungal Shannon index and richness with microbial *T_min_*, mean annual temperature (MAT), pH, soil organic matter (SOM), and mean annual precipitation (MAP). Significant regressions are in bold.

|  | **Bacterial** | | | | **Fungal** | | | |
| --- | --- | --- | --- | --- | --- | --- | --- | --- |
|  | **Shannon index** | | **Richness** | | **Shannon index** | | **Richness** | |
|  | ***R^2^*** | ***p*** | ***R^2^*** | ***p*** | ***R^2^*** | ***p*** | ***R^2^*** | ***p*** |
| **Microbial** *T_min_* | 0.13 | **0.002** | 0.15 | **<0.001** | 0.02 | 0.21 | 0.02 | 0.21 |
| **MAT** | 0.09 | **0.01** | 0.13 | **0.002** | 0.01 | 0.70 | 0.00 | 0.86 |
| **pH** | 0.27 | **<0.001** | 0.36 | **<0.001** | 0.06 | 0.05 | 0.08 | **0.01** |
| **SOM** | 0.11 | **0.004** | 0.14 | **0.001** | 0.06 | 0.06 | 0.05 | 0.06 |
| **MAP** | 0.01 | 0.52 | 0.01 | 0.78 | 0.01 | 0.77 | 0.00 | 0.86 |

**Table S7.** Bacterial and fungal ASVs that correlated significantly with *T_min_* values. Warm-adapted taxa correlated positively with *T_min_*, while cold-adapted taxa correlated negatively. Only the ten most correlated taxa for warm- or cold-adapted bacterial and fungal taxa are shown (see Appendix 1 for the full list). Bacterial ASVs were examined with the database Microbe Atlas to identify their prevalence and fungal ASVs were searched in the Global Fungi database to identify the most frequent temperature where they have been reported (see Supplementary methods).

Continued Table S7

**Table** **S8.** Results from the *envfit* analyses of the bacterial and fungal communities with environmental data. *R^2^*-values are the squared correlation coefficient and *p*-values are based on random permutations of the data. Significant correlations are in bold.

|  | **Bacterial NMDS envfit** | | **Fungal NMDS envfit** | |
| --- | --- | --- | --- | --- |
|  | ***R^2^*** | ***p*** | ***R^2^*** | ***p*** |
| **pH** | 0.60 | **0.001** | 0.57 | **0.001** |
| **SOM** | 0.48 | **0.001** | 0.43 | **0.001** |
| **Longitude** | 0.05 | 0.154 | 0.07 | 0.085 |
| **Latitude** | 0.34 | **0.001** | 0.48 | **0.001** |
| **MAT** | 0.36 | **0.001** | 0.54 | **0.001** |
| **MAP** | 0.05 | 0.205 | 0.09 | **0.043** |
| **Microbial *T_min_*** | 0.17 | **0.002** | 0.24 | **0.001** |
